# Dysregulation of the serine/threonine phosphatase PP2A contributes to autoimmunity

**DOI:** 10.1186/ar3964

**Published:** 2012-09-27

**Authors:** JC Crispín

**Affiliations:** 1 Beth Israel Deaconess Medical Center, Harvard Medical School, Boston, MA, USA

## 

Protein phosphatase 2A is a ubiquitously expressed serine/threonine phosphatase that regulates a large number of cellular processes. Levels of the catalytic subunit of PP2A (PP2Ac) are tightly controlled. T cells from patients with systemic lupus erythematosus (SLE) express abnormally high levels of PP2Ac. This is promoted by a lupus-associated SNP and by DNA hypomethylation that alters local transcription factor binding. PP2Ac regulates CREB, Elf-1, and SP1, and could thus contribute to several molecular abnormalities described in SLE T cells. In order to determine whether increased levels of PP2Ac in T cells play a role in the development and/or promotion of autoimmune disease, we genetically engineered a mouse to transgenically express high levels of PP2A in T cells. This isolated abnormality allowed us to evaluate the effects of PP2Ac dysregulation in an otherwise normal immune system.

PP2Ac transgenic (Tg) mice developed modest splenomegaly and lymphadenopathy, but no overt signs of autoimmunity. Susceptibility of PP2Ac Tg mice to immune-mediated disease was evaluated by antibody-induced glomerulonephritis. Glomerular damage and proteinuria were significantly more severe in PP2Ac Tg mice than in nontransgenic littermates. To determine the mechanism underlying increased kidney damage, we analyzed cytokine production by T cells from PP2Ac Tg and control mice. Production of IL-17 was increased ~10-fold in Tg mice. This phenomenon was documented *in vitro *and *in vivo *and was pathologically relevant since blockade of IL-17 abrogated the enhanced susceptibility to glomerulonephritis of Tg mice. These results indicated that increased PP2Ac levels enhance the production of IL-17 in T cells and, through this mechanism, promote immune-mediated organ damage. To determine the molecular pathways through which PP2Ac enhances IL-17 production, we analyzed differentiation of CD4 T cells into effector subsets (that is, Th1, Th2, Th17). Although IL-17A and IL-17F production were constantly higher in PP2Ac Tg T cells, other Th17 characteristics did not differ between Tg and control T cells suggesting that the IL-17-inducing effect occurred independently of Th17 differentiation. Analysis of the *Il17 *locus demonstrated that PP2Ac overexpression was associated with local permissive epigenetic changes (increased histone 3 acetylation), which explained enhanced IL-17 production.

We provide evidence that supports dysregulation of PP2Ac in T cells is able to facilitate autoimmune disease by promoting the production of IL-17. This effect is independent of Th17 differentiation and is explained by the capacity of PP2Ac to modify chromatin accessibility at the *Il17 *locus by inducing defined epigenetic modifications.

